# Modern diet and metabolic variance – a recipe for disaster?

**DOI:** 10.1186/1475-2891-13-15

**Published:** 2014-02-06

**Authors:** James P Grantham, Kaspar Staub, Frank J Rühli, Maciej Henneberg

**Affiliations:** 1 School of Medical Sciences, The University of Adelaide, Lincoln College, 45 Brougham Place, Adelaide 5006, SA, Australia; 2 Centre for Evolutionary Medicine, Institute of Anatomy, University of Zürich, Zürich, Switzerland

**Keywords:** Alanine transaminase, Body mass index, Adiposity, Swiss, Diet

## Abstract

**Objective:**

Recently, a positive correlation between alanine transaminase activity and body mass was established among healthy young individuals of normal weight. Here we explore further this relationship and propose a physiological rationale for this link.

**Design:**

Cross-sectional statistical analysis of adiposity across large samples of adults differing by age, diet and lifestyle.

**Subjects:**

46,684 19–20 years old Swiss male conscripts and published data on 1000 Eskimos, 518 Toronto residents and 97,000 North American Adventists.

**Measurements:**

Serum concentrations of the alanine transaminase, post-prandial glucose levels, cholesterol, body height and weight, blood pressure and routine blood analysis (thrombocytes and leukocytes) for Swiss conscripts. Adiposity measures and dietary information for other groups were also obtained.

**Results:**

Stepwise multiple regression after correction for random errors of physiological tests showed that 28% of the total variance in body mass is associated with ALT concentrations. This relationship remained significant when only metabolically healthy (as defined by the American Heart Association) Swiss conscripts were selected. The data indicated that high protein only or high carbohydrate only diets are associated with lower levels of obesity than a diet combining proteins and carbohydrates.

**Conclusion:**

Elevated levels of alanine transaminase, and likely other transaminases, may result in overactivity of the alanine cycle that produces pyruvate from protein. When a mixed meal of protein, carbohydrate and fat is consumed, carbohydrates and fats are digested faster and metabolised to satisfy body’s energetic needs while slower digested protein is ultimately converted to malonyl CoA and stored as fat. Chronicity of this sequence is proposed to cause accumulation of somatic fat stores and thus obesity.

## Introduction

One of the greatest challenges facing the future of human health stems from our aptitude in procuring sustenance as more individuals become overweight or obese. Excessive weight has long been accepted as a by-product of an indulgent lifestyle and was even thought of as a marker of affluence in many societies [1]. However, in a post-Framingham world, obesity has become a prelude to adverse health and premature death [2]. The World Health Organisation estimates that obesity contributes significantly to the disease burdens of, among others, diabetes (44%), ischaemic heart disease (23%) and carcinogenesis (7-41%) [3]. Moreover, those considered overweight or obese have been subject to discrimination and prejudice [4].

Body weight status is determined with reference to the body mass index. Those with a BMI ranging between 18-24.99 kg/m^2^ are considered healthy. Individuals falling within the 25.00-29.99 kg/m^2^ category are classified as overweight whilst obesity is reserved for those reaching or exceeding a body mass index of 30 kg/m^2^[5]. Despite of all the disincentives, the issue of weight has accelerated towards becoming a universally prominent health concern, particularly in Western societies. As of 2008, approximately 35% of the global adult population were overweight whilst 10% of males and 14% of females were considered obese [6]. This figure is much higher in affluent regions, including the Americas (62% and 26% respectively) [7] and Australia (35% and 28% respectively) [8].

The majority of research into obesity has focused directly on the caloric imbalances. The results of such investigations have led to the development of the central dogma of obesity. The dogma, repeated in textbooks, stipulates that if energy input outstrips energy expenditure, increased weight ensues as excess energy is converted into triglycerides and shuttled into adipose tissue [9]. Clearly, this mechanism is a prominent contributor in the development of obesity but the appeal of a simplistic causation and the subsequent holistic acceptance afforded to the theory has diminished exploration of alternative views. Indeed, if the aetiology of obesity was solely attributable to lifestyle factors then the trend would be reversible through caloric restriction and physical exertion. However, contrary to popular belief, the majority of contemporary methods of weight loss prove ultimately unsuccessful [1]. The individual response variation in terms of weight loss despite apparently equivalent energy intake and expediture warrants further investigation. The inference from these inconsistencies is that there are multiple driving factors in the aetiology of weight gain.

In recent years, alternative hypotheses have been explored. Scientists at the Pennington Biomedical Research Centre have implicated adenovirus-36 infection in the genesis of obesity [10]. The virus is speculated that this leads to a proliferation of fatty tissue through the induction of cellular differentiation [10]. The role of bacterial gut flora constituents is also known to influence intestinal absorption of nutrients [11]. Meanwhile, several studies have incriminated sleep deprivation in the development of excess body mass, citing disruptions in glucose metabolism, appetite regulation and energy expenditure [12]. Some recent investigations have even focused around national soy consumption and obesity rates. Authors of this finding suggested that the xenoestrogens present within the product impact on thyroid function [13].

The *European Heart Journal* recently published an article outlining two broad profiles of obese patients. It was found that the majority of patients with obesity were, as to be expected, metabolically unhealthy [14]. However, approximately 46% of the obese cohort was classified as metabolically healthy and possessed fitness levels and mortality comparable to their non-obese counterparts [14]. Consequently, this has led to speculation that separate mechanisms may be driving the different obesity phenotypes. One such hypothesis is that a positive energy balance drives metabolically healthy individuals towards obesity whilst innate metabolic deregulation is present within the majority of obese individuals. Perhaps relaxed natural selection is involved through the accumulation of genes coding suboptimal energy metabolism [15]. This view is supported by the presence of obesity within malnourished populations in which a traditional positive energy balance scenario would seem unlikely [16].

The growing trend towards a personalised medical approach has led to a greater appreciation of variant expression of a disease because of anatomical or metabolic variation. The issue of weight control may also exhibit such inconsistencies. Recent evidence has suggested that body frame variation may play an integral role in obesity development. Those with greater lean trunk volume have been found to have thicker layers of adipose tissue [17]. The speculations surrounding such a phenomenon stem from the larger abdominal viscera present within these subjects, suggesting greater gastric distensibility or intestinal absorption. The importance of metabolic variation has been highlighted by the predictive capacity of the enzyme alanine transaminase (ALT) with regard to body weight. A recent study concluded that ALT was more closely associated with bodyweight than cholesterol, blood pressure and random blood glucose levels [18].

A broad study of the relevant data is necessary to ascertain new potential causations for the obesity epidemic. In the interests of this paper, data were used from various sources accessed from personal archives and extensive internet searches.

## Materials and methods

Data were collated from previously used sources, primarily from 101,392 Swiss conscripts from all national cantons [19,20]. Only males aged 19 or 20 with complete information regarding all variables were included for the purposes of analysis, thus reducing the sample size to 46,684 individuals. Swiss armed forces require compulsory enlistment over the course of several days. During this period, medical and performance testing was conducted under appropriate medical supervision within six fully accredited centres across all major Swiss geographical regions. Individual occupation as well as predominant language and religion within each individual’s municipality were recorded in an attempt to control economic and cultural influences. Extensive laboratory analysis incorporated blood samples. All of the individuals in the study were made completely anonymous prior to analysis. Under provisions of the Swiss Data Privacy act (SR235.1;19.6.1992) no separate ethical approval is required for anonymized statistical data held by the Swiss Government. Since reporting for conscription is a primary duty of each male Swiss citizen, and those citizens are thoroughly informed about this duty prior to conscription, their participation is automatically considered an informed consent. Further details can be found in [18].

An in-depth internet search was conducted for data relating to various dietary restrictions and the subsequent outcomes on body mass index. For the purposes of illustrating contrast, a modern society known to consume limited carbohydrates and considerable amounts of protein was sought. These criteria were met by the native Inuit of the Central and Eastern Canadian Arctic [21]. This population had been subject to extensive anthropometric analysis with a particular interest in their weight. A study conducted between 1964 and 1970 with more than 1000 participants was selected for analysis [22]. To contrast this with the appropriate non-tailored dietary sample, a study on skin-fold measurements amongst 518 Toronto residents from 1969 was used [23].

Information regarding the remainder of the spectrum, namely vegetarians with their high carbohydrate, low protein diet and the unrestricted omnivorous populations were satisfied by a study on the health status of 97,000 Adventist Church members across the U.S and Canada [24]. Participants were classified based on the nature of their diet, including vegans, varying degrees of vegetarianism and non-vegetarians. Many potential confounding variables including education level, socio-economic status, physical activity level, and sleep quantity were recorded and controlled for as much as possible. For the purposes of this study, efforts were focused on the relationship between dietary nature and body mass index.

Statistical analysis of the data was carried out using SPSS Statistics 19.0 and Microsoft Excel. The schematic diagram within the text that illustrates the mechanism of the alanine cycle was constructed using Microsoft Paint.

## Results

As can be seen in Table 1, even when using all participants in the Swiss study, there was a very significant partial correlation co-efficient linking individual ALT levels and body mass index, despite controlling for post-prandial glucose levels. Values of correlation co-efficients shown in this table indicate that ALT is responsible for approximately 10-15% of variance in body mass index. Stepwise multiple regression conducted on the sample (results not shown) confirmed that ALT accounts for approximately 14% of the total variance in body mass using conservative measurements. The reality is likely to be much higher given the measurement error for physiological assessments ranging between 30 and 40% for many parameters [25]. If a scenario was accepted in which only a 70% correlation was possible, given measurement variance, the enzyme may, in reality, account for up to 28% of the individual variance.

**Table 1:** The statistical correlation between ALT and BMI with and without controlled variables

**Group selected**	**Number**	**Spearman correlation**	**Variables kept statistically constant**	**Partial Pearson correlation**
All		0.395	1. Post-prandial glucose levels	0.370
	46182	P = 0.000		P = 0.000
All	44714	0.395	1. Post-prandial glucose levels	0.314
		P = 0.000	2. Blood pressure	P = 0.000
			3. Cholesterol	
			4. Leukocytes	
			5. Thrombocytes	
"Metabolically healthy"		0.237	1. Post-prandial glucose levels	0.215
1. Systolic Blood Pressure < 120 mmHg	1341	P = 0.000		P = 0.000
2. Glucose < 7.8 mmol/L				
3. Cholesterol < 5.0 mmol/L				
4. Leukocytes < 7100/μL				
5. Thrombocytes < 260,000/μL				
"Metabolically healthy" but non-obese	1329	0.227	1. Post-prandial glucose levels	0.194
1. Systolic Blood Pressure < 120 mmHg		P = 0.000		P = 0.000
2. Glucose < 7.8 mmol/L				
3. Cholesterol < 5.0 mmol/L				
4. Leukocytes < 7100/μL				
5. Thrombocytes < 260,000/μL				
6. Body mass index < 30 kg/m^2^				

Traditionally, elevated ALT levels within overweight or obese individuals have been considered a marker of hepatocellular damage, often associated with non-alcoholic fatty liver disease [26]. In order to test this belief, with further reference to Table 1, the sample was then controlled for all other markers of metabolic dysfunction, including glucose, blood pressure, cholesterol, leukocytes and thrombocytes [27]. The results reveal that there remains a significant correlation between ALT and body mass. On this occasion, conservative estimates suggest that ALT accounts for approximately 10% of individual variance in body mass index. Furthermore, all participants in the study were young males with less than 5% considered obese. The conclusion is that it is highly unlikely that these individuals would show marked liver damage, making it reasonable that ALT must now be entertained as a potential causative factor of weight gain rather than simply a collateral factor.

Even when selecting the 10% of the metabolically healthiest individuals, the partial correlation between ALT and body weight remains very statistically significant (Table 1). Similar results were obtained irrespective of whether only non-obese individuals or the entire sample that fits the criteria are selected.

**Figure 1:**
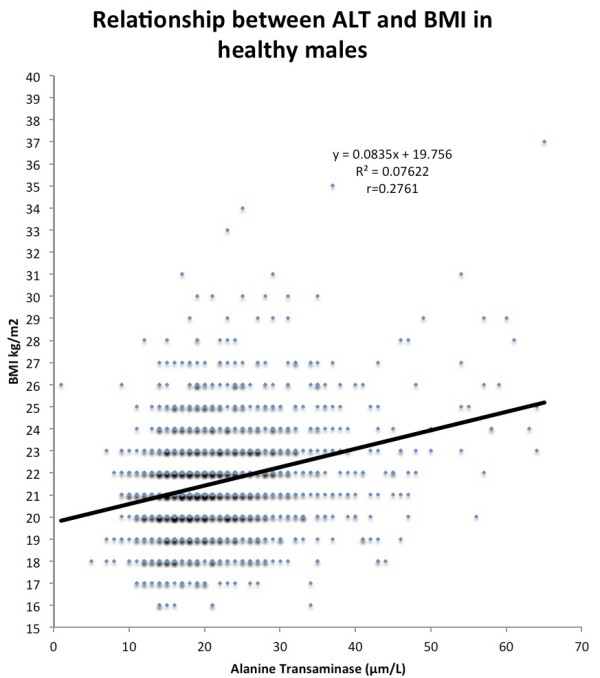
The relationship between ALT and BMI in healthy young Swiss males.

Figure 1 is a graphical representation of the relationship between ALT and Body Mass Index. The slope was obtained using linear regression and demonstrates the relationship between ALT and body mass index. As shown in the image, there is a clear link between an individual’s level of the enzyme and body weight. Further analysis was conducted based on this finding in order to extrapolate its significance. It was determined that if an individual possessed levels of the enzyme one standard deviation above the average, its influence may increase the BMI of that individual by 3.59 kg/m^2^. Under the same conditions, an individual with ALT levels at two standard deviations above the population mean will experience a 5.01 kg/m^2^. The following equation can be applied to express the aforementioned relationship: Y = 19.756 + 0.0835(ALT).

**Figure 2:**
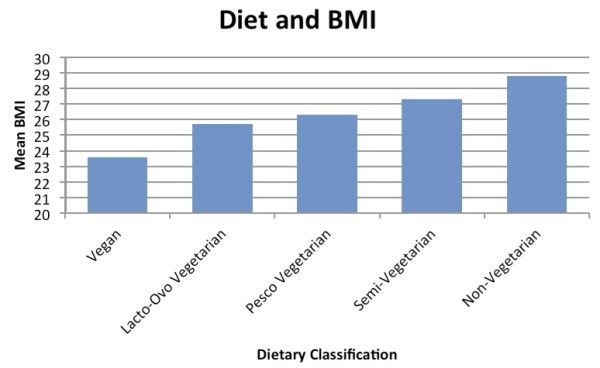
**Average BMI across diets.** A survey of ~100,000 members of the Seventh Day Adventist Church in North America [24].

**Figure 3:**
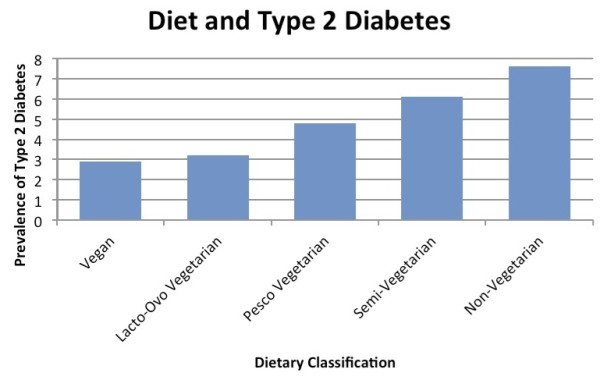
**Prevalence of diabetes across diets.** A survey of ~100,000 members of the Seventh Day Adventist Church in North America [24].

**Figure 4:**
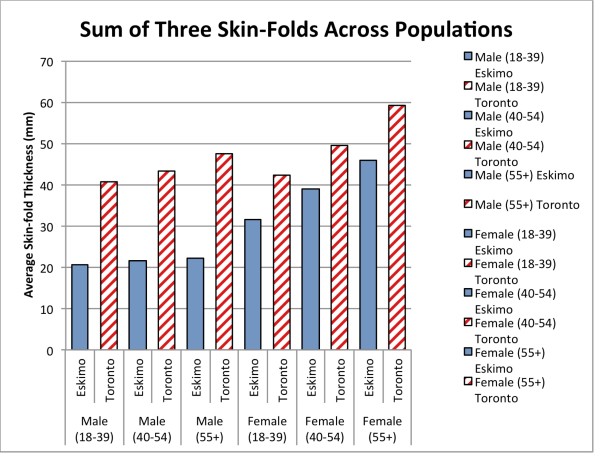
**Average sum of three skin-fold measurements of various age categories and sexes from Toronto residents and Canadian Inuit.** Data collected in 1960’s and 1970’s. – Sources [21-23].

Figures 2 and 3 show the comparative rates of obesity and diabetes respectively across various diets in North American Adventists. Figure 2 illustrates the relationship between body mass index and the type of diet. Those individuals that consume a vegan diet possessed an average body mass index of 23.6 kg/m^2^. The classic form of vegetarianism, the lacto-ovo vegetarian showed an average BMI of 25.7 kg/m^2^. Simultaneously, the cohort with an unrestricted consumption of animal products revealed an average body mass index of 28.8 kg/m^2^. Similarly, Figure 3 outlines the three-fold discrepancy in type 2 diabetes mellitus between vegans and non-vegetarians. Figure 4 represents a comparison of skin-fold measurements recorded between 1964 and 1970 from native Canadian Inuit and Toronto residents. It also highlights the difference in body mass between the two populations. The discrepancy between males from different environments is far more pronounced, with the sum of skin folds effectively doubled amongst the Westernised citizens of Toronto. In females the difference was less, but still significant.

## Discussion

### Quality and reliability of results

Amongst the Swiss conscripts, no manipulation of the data was conducted in achieving the gross relationship between ALT and obesity. However, in the interests of further eliminating the possibility of raised ALT levels being a by-product of obesity related liver damage that the metabolically healthy individuals were selected as a separate sample for further analysis. The relatively large sample size and the simplicity of analysis reduces the scope for bias or type II statistical error.

To illustrate the essential hypothesis of the paper, data regarding levels of obesity across varying dietary protein constraints were actively sought out. Ultimately, the sample chosen came from a study conducted in the infancy of this century comprised over 60,000 participants. The results of the study are particularly reliable given that all volunteers shared a commonality through their religious belief and geography (Adventists in North America). Additionally, various confounding factors were considered in the analysis including level of education, income and physical activity.

The final information included within the paper was compiled from multiple sources documenting skin-fold measurements of Canadians. The results were taken from samples collected in the 1960’s for both Canadian Eskimos and European origin residents of Toronto. With such a comparison, the results are best taken as a guiding trend rather than absolute confirmation.

### Hypothesis

Through the procedures outlined above, we have observed a significant relationship between individual ALT levels and body mass index. Such a correlation suggests a metabolic maladaptation to current dietary habits. An evolutionary mismatch between modern dietary constituents and the food available prior to the agricultural revolution has long been considered a factor in the obesity epidemic [28].

Traditional conceptualisations of obesity have typically ignored the role of individual human variation, favouring a simplistic energy input, energy expenditure approach. However, the importance of genetic tendencies is attracting scientific attention. A study conducted in 1990 showed that adopted individuals in later life demonstrated a greater conformity in body weight to their biological parents than their adopted parents [29]. This emphasises the importance of heritable influences over environmental factors acting later in life. Multiple studies have illustrated the variability in both weight loss and gain across individuals despite controlling for dietary intake and exercise.

A well-documented example of metabolically driven weight gain exists physiologically. When females approach menarche, their bodies attempt to conserve energy via the development of adequate adipose tissue reserves. This metabolic phenomenon has also evolved to accumulate enough energy stores that in the event of conception, adequate resources would be available to sustain pregnancy [30]. The increase in adiposity often occurs in conditions otherwise rarely conducive to weight gain, such as limited dietary intake experienced by the rural Cape Coloured youth of the Klein Karoo district [31].

It is likely that metabolic processes that drive weight gain are multiplicative, employing a variety of different pathways of energy metabolism. An earlier paper published on this topic identified the hepatic enzyme, alanine transaminase, as a strongest predictor of body mass index of all observed variables [18]. It is, therefore, prudent to consider ALT as one such method of metabolic weight regulation.

**Figure 5:**
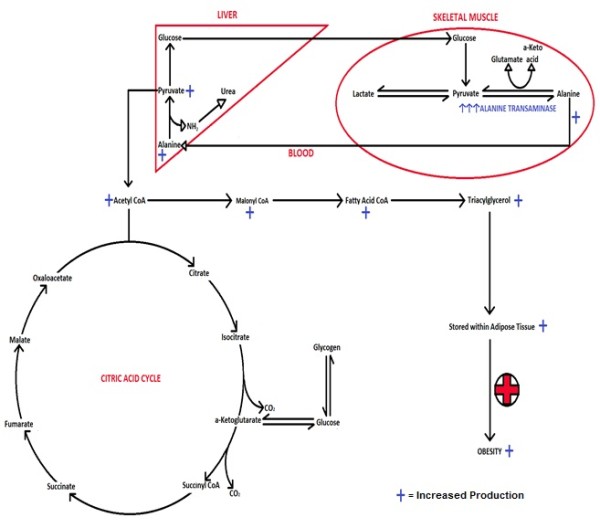
The mechanism of ALT increasing gluconeogenesis and lipogenesis (constructed by authors).

ALT is central in protein metabolism, with specific reference to its role in gluconeogenesis [32]. As Figure 5 illustrates, alanine transaminase, is the enzyme responsible for reversibly converting alanine to pyruvate. Subsequently, pyruvate can be converted to acetyl CoA. This substrate can either enter the Krebs cycle for the production of energy or be shunted down a lipogenic pathway to be stored within adipocytes depending on the imminent energy demands.

From this knowledge, it is possible to deduce that individuals with higher levels of the enzyme may be more efficient at alanine recycling. In these individuals, it seems likely that a greater portion of the substrate, in this case alanine, is reversibly converted back into pyruvate. This may result in a greater input of pyruvate into the citric acid cycle, or, in the event of sufficient immediate energy stores, a greater conversion into malonyl CoA. This suggests that individuals endowed with elevated transaminase levels will more effectively mobilise alanine in the interests of gluconeogenesis and fat deposition. Naturally, weight gain will ensue. A similar situation may exist with regard to other transaminases producing carbon skeletons of other amino acids.

Furthermore, other links were able to be made to weight regulation and alanine transaminase. As shown in Figure 5, a product of converting alanine into pyruvate is glutamate. Previous research indicates that elevated levels of glutamate derived from dietary intake can be toxic to the appetite-regulating centres of the brain, namely the arcuate nucleus within the thalamus [33]. It is therefore reasonable that a metabolically driven accumulation of glutamate may also have neuronal implications. Perhaps in these individuals, a synergistic down-regulation in appetite suppression results in increased caloric intake and contributes to the gradual accumulation of adipose tissue.

### Detracting arguments

Intuitively, if a protein rich diet causes weight loss then eating protein should not increase body weight. This assertion is correct in the absence of abundant carbohydrates because energy derived from protein consumption would be expended through metabolising the amino acids. However, if a rich food source, containing both carbohydrates and proteins, were to be ingested then the energy from the carbohydrates may be power protein metabolism. The key feature is that energy is more readily elicited from carbohydrates than protein [34]. The result of such a process is an increased net yield from the protein source, which, in the absence of any immediate energy requirements, may be converted into fats and stored within adipocytes.

Another contention is that the elevated levels of ALT are often considered a marker of liver disease, often associated with obesity. The resultant inference is that alanine transaminase ALT may be correlated with increased weight due only to hepatic dysfunction. However, this is unlikely to apply to more than a small minority of the participants for a number of reasons. First and foremost, the age of all participants analysed fell between 19 and 20, quite young for liver disease. Furthermore, the majority of the individuals tested fell within a healthy weight range, with only approximately 5% of the participants classified as being obese. Finally, even when eliminating those considered obese and those with any markers of metabolic dysfunction, a clear and significant correlation remains. It is therefore, unlikely that the superfluous weight is solely responsible for inducing heightened enzymatic recordings.

The final criticism of the hypothesis relates to the synergistic factors that conspire towards weight gain. Research indicates a number of the markers related to obesity are often inter-related. For example, vegan and vegetarian diets are often associated with higher education and socioeconomic status [35]. These two characteristics typically mitigate weight gain through healthier lifestyle choices, independent of dietary factors [36]. Therefore, it is easy to dismiss the importance of the dietary constraints emphasised within that group. However, in the study from which the data were retrieved, many of these confounding factors were incorporated into the questionnaire provided. Interestingly, the vegans and non-vegetarians showed similar degrees of education, socioeconomic distribution and participation in physical activity. Therefore, it was possible to illustrate that even in the absence of prominent confounding factors, dietary habits still had a substantial effect.

### Evolutionary perspective

The physiological grounding for our hypothesis has its roots in the evolutionary process. Humans belong to the mammalian animal class, a group that primarily derives nutrients from fatty acids and proteins [37]. Resultantly, humans are inept to deal with efficient storage of simple sugars with storage depots for glycogen present only within the liver and skeletal muscle [38]. Even at maximum capacity, the energy derived from stored glycogen is sufficient for only one day worth of activity [39]. Conversely, the potential energy reserves mobilised from adipose tissue may be sufficient to sustain life for a number of weeks in most individuals. Additionally, human metabolism is such that it is much more adept at converting excess protein into fat than converting superfluous sugars into fats for long-term storage [40]. These features have developed because it was much more commonplace within the environment of evolutionary adaptation to eat a slain animal periodically than consume an abundant source of carbohydrates in the single sitting.

Our ancestors adapted to increased reliance on meat as our gastrointestinal system attests [41-43]. Animals with carnivorous tendencies often derive their energy requirements from animal fats and proteins through efficient metabolic systems. Ingested fats, followed by simple sugars are the simplest to break down and utilise for energy production for somatic use [44]. Conversely, protein digestion is more laborious and time consuming before the carbon skeletons of amino acids can be used in the citric acid cycle which provides energy to all somatic cells [45]. The ingestion of simple sugars and easily digestible fats results in a rapid spike in the metabolic rate [46].

Coevally, the rarity of sugars across the natural landscape of evolution has driven the cravings for these foods once considered delicacies [47]. Traditionally, they appear in considerable quantities only in the nectar attracting pollinating animals [48]. However, through the domestication of plants and animals, humans have placed an increasing emphasis on the production of simple sugars and starch. The milking of various domesticated animals has yielded a sugar and fat rich dietary staple for many pastoralist communities and developed nations [49]. Additionally, the agricultural production of grain now provides the bulk of food resources in all major cultures with the supplementation of fruits and vegetables.

Therefore humans are the only large mammal that derives majority of its body energy by absorbing and metabolising sugars. Because sugar metabolism primarily concentrates on the oxidation of carbohydrates in the direct production of energy, this rarely produces fat [50]. Therefore, as long as protein and fat sources are ingested in modest quantities, even an excessive consumption of carbohydrates will not markedly contribute to fat deposition.

However, in a situation through which the caloric requirements are met completely by simple sugars and starches, concurrent with consumption of other foodstuff, danger presents. In such an environment, the consumption of proteins will result in the conversion of protein into triglycerides. This process, if continued, will lead to significant storage of these molecules within adipose tissue and thus, obesity. This, in part, is due to the fact that there is no reservoir for protein storage within the body to be used as a regular source of energy [51]. Therefore, in normal circumstances, any amino acids which are not immediately required for structural, enzymatic or hormonal bodily functions will be readily deaminated. The resulting short carbon skeletons will then be converted into fats for long-term storage [52].

### Modern implications

If accepted, the implications of the hypothesis presented may be significant. The current understanding of weight gain is a simple relationship between energy intake and expenditure, with little other consideration. With the role of ALT elucidated, it is possible to appreciate just one way in which individual human variation contributes to the obesity epidemic. The way of the future seems to lie in expanding biological and social understanding of obesity so that it is possible to target specific triggering factors for particular individuals.

Another important conclusion to be made upon accepting this hypothesis is the necessity for the dietary guidelines to be reconsidered. As it stands currently, the basis of nutritional directives centre around a balanced meal plan in which all dietary constituents are apportioned appropriately. The prototype of current guidelines is often taught around and derived from the classic food pyramid. Perhaps consequently, the desire for a balanced diet has permeated into the social cuisine both at home and at restaurants with many meals incorporating both proteins and carbohydrates. In accordance with the aforementioned hypothesis, this combination may be more deleterious than beneficial. If our conclusions are ultimately affirmed, a more salubrious diet would seek to divorce carbohydrates and proteins from the same plate.

### Future considerations

The new information regarding ALT may be used in the global fight against obesity epidemic. It seems likely that if the causative relationship between ALT and weight gain is substantiated, and that the next step would be individual testing of the enzyme’s levels. In theory, it may be practical to screen young members of population in order to identify those with heightened levels of alanine transaminase, identifying those metabolically predisposed to obesity. The prudent management of the individual would be to tailor a targeted program designed to prevent adiposity, thereby circumventing a heavy future.

The other foreseeable approach is to directly target the levels of the transaminase enzyme itself. If technology permits a future in which it is safe and effective to manipulate concentrations of ALT, it may be possible to eliminate elevated levels as a gluconeogenic factor leading to obesity. Future medical therapies may prove capable of producing weight loss or preventing weight gain.

The final avenue of approach may reside within prescribing specialised diets that separate proteins and carbohydrates. It may become part and parcel of futuristic dieting to alternate between herbivorous and carnivorous meals, with omnivorous diets being infrequently used. These eating habits would hark back to the evolutionary origins of the human species and may prove the best way forward. It is often observed that by understanding our human past, this may best inform our future.

## Competing interests

The authors declare that there are no conflicts of interest, financial or otherwise, related to this paper.

## Authors’ contributions

JG drafted the text with contributions from MH, JG and MH formulated the hypothesis together. FR and KS obtained the data, conducted preliminary analyses and provided suggestions for the hypothesis. MH performed the final statistical analysis and JG constructed figures. All authors reviewed, edited and approved the final manuscript.
